# How to give cells an identity crisis

**DOI:** 10.7554/eLife.112549

**Published:** 2026-08-13

**Authors:** Paige Dillon, Julie Hollien

**Affiliations:** 1 School of Biological Sciences and the Center for Cell and Genome Science, University of Utah Salt Lake City United States

**Keywords:** cell identity, CHOP, stress, ER, chronic, acute, Human

## Abstract

The transcription factor CHOP helps cells switch from an emergency stress response to a chronic one, where cells survive but lose some of the functions that define their identity.

**Related research article** Velarde TF, Liu K, Zhang Z, Adajar RC, Zhao C, Cao H, Rutkowski DT. 2026. CHOP promotes the transition to chronic integrated stress response signaling with suppression of hepatocyte identity. *eLife* **15**:RP112049. doi: 10.7554/eLife.112049.

For humans, stress is often an unavoidable part of life that can affect us down to the cellular level. Cellular stress can arise from physiological fluctuations, such as changes in nutrient or metabolite concentrations, as well as from toxins, infection or disease.

Cells rely on sophisticated protective pathways that help them detect stress, adapt and survive. In the face of persistent, chronic stress, cells sometimes die through apoptosis. This form of programmed cell death has long been viewed as both a failure of the protective pathways to resolve the stress and a potential underlying cause of chronic disease. Now, in eLife, Thomas Rutkowski and colleagues at the University of Iowa and the Shanghai Cancer Institute – including Theo Veralde as first author – report new insights on cellular stress that challenge this view (Velarde et al., 2026).

One of the most extensively studied forms of cellular stress originates in one of the cell’s own organelles: the endoplasmic reticulum (ER). As the entry point for the secretory pathway, the ER is a highly effective protein-folding compartment and, in some cells, can support mind-boggling rates of protein secretion, such as the 15 million molecules of serum albumin estimated to be secreted by a single liver cell every minute (Schulze et al., 2019). Despite these impressive feats, the ER can be overwhelmed by rapid increases in newly synthesized proteins or by pathologies that affect protein folding or trafficking. This imbalance between the capacity and load on the ER is the classic definition of ER stress, which is linked to a variety of diseases including neurodegenerative diseases, cancer, diabetes and cardiovascular disease (Acosta-Alvear et al., 2025).

Veralde et al. focused on the transcription factor CHOP, which promotes cell death when the ER is under stress. CHOP also seems to be important during mild and chronic stress, although it does not always induce cell death under these conditions (Liu et al., 2024). To study the effects of CHOP in mice, the researchers used liver cells engineered to lack the transcription factor and challenged them with the ER stress inducer tunicamycin.

Deletion of CHOP altered the way liver cells responded to stress, with one of the most prominent effects being reduced steatosis, or lipid accumulation. Through a combination of RNA-seq and ChIP-seq experiments, Verlade et al. showed that CHOP directly suppresses genes that maintain the normal identity and metabolic functions of liver cells; one such gene is ONECUT1, a central transcription factor regulating liver-specific metabolism and cell identity. Many of ONECUT1’s targets were also suppressed, supporting the conclusion that CHOP downregulates liver cell identity and metabolism through ONECUT1.

In the absence of CHOP, these cells continued with their normal metabolism program, explaining the reduced steatosis in mice lacking CHOP. This seems to fit nicely with the idea that chronic disease might not be a direct consequence of irremediable stress and cell death, but rather a consequence of the successful cure for stress resulting in a loss of cell identity, leading to secondary, detrimental long-term effects.

A second main finding is that CHOP prolongs the cell’s stress-induced gene expression patterns while turning off the emergency response. Normally, when the ER becomes overloaded with misfolded proteins, cells activate an unfolded protein response (Wiseman et al., 2022), which temporarily slows protein production, expands the ER’s protein-folding capacity, and removes misfolded proteins. Part of this response involves phosphorylation of the translation initiation factor eIF2α (Ryoo, 2024), which reduces global protein production and gives the ER time to recover. At the same time, eIF2α phosphorylation triggers a stress-responsive gene expression programme.

The work of Velarde et al. suggests that CHOP helps keep protective stress-signalling pathways active, even after eIF2α phosphorylation has begun to decline and protein synthesis is being restored. This prolonged response resembles a shift from an acute to a chronic stress state and disrupts fat metabolism in the liver. This indicates that CHOP helps cells adapt to ongoing stress, but at the cost of altering the gene programmes that preserve their normal identity and function. The efficiency by which cells restore translation while resetting to their gene expression programme is likely a key determinant in how cells adapt to chronic stress without losing their identity.
